# The urine albumin-to-creatinine ratio is a reliable indicator for evaluating complications of chronic kidney disease and progression in IgA nephropathy in China

**DOI:** 10.6061/clinics/2016(05)01

**Published:** 2016-05

**Authors:** Lu Huan, Luo Yuezhong, Wang Chao, Tu HaiTao

**Affiliations:** I Guangzhou University of Traditional Chinese Medicine, Guangzhou, Guangdong, PR, China; II The First Affiliated Hospital of Guangzhou University of Traditional Chinese Medicine, Department of Nephrology, Guangzhou, Guangdong, PR, China

**Keywords:** Albuminuria, Chronic Kidney Disease, Glomerulonephritis, IgA Nephropathy, Proteinuria

## Abstract

**OBJECTIVE:**

This study investigated the correlation between the albumin-to-creatinine ratio in the urine and 24-hour urine proteinuria and whether the ratio can predict chronic kidney disease progression even more reliably than 24-hour proteinuria can, particularly in primary IgA nephropathy.

**METHODS:**

A total of 182 patients with primary IgA nephropathy were evaluated. Their mean urine albumin-to-creatinine ratio and 24-hour proteinuria were determined during hospitalization. Blood samples were also analyzed. Follow-up data were recorded for 44 patients. A cross-sectional study was then conducted to test the correlation between these parameters and their associations with chronic kidney disease complications. Subsequently, a canonical correlation analysis was employed to assess the correlation between baseline proteinuria and parameters of the Oxford classification. Finally, a prospective observational study was performed to evaluate the association between proteinuria and clinical outcomes. Our study is registered in the Chinese Clinical Trial Registry, and the registration number is ChiCTR-OCH-14005137.

**RESULTS:**

A strong correlation (r=0.81, *p*<0.001) was found between the ratio and 24-hour proteinuria except in chronic kidney disease stage 5. First-morning urine albumin-to-creatinine ratios of ≥125.15, 154.44 and 760.31 mg/g reliably predicted equivalent 24-hour proteinuria ‘thresholds’ of ≥0.15, 0.3 and 1.0 g/24 h, respectively. In continuous analyses, the albumin-to-creatinine ratio was significantly associated with anemia, acidosis, hypoalbuminemia, hyperphosphatemia, hyperkalemia, hypercholesterolemia and higher serum cystatin C. However, higher 24-hour proteinuria was only associated with hypoalbuminemia and hypercholesterolemia. Higher tubular atrophy and interstitial fibrosis scores were also associated with a greater albumin-to-creatinine ratio, as observed in the canonical correlation analysis. Finally, the albumin-to-creatinine ratio and 24-hour proteinuria were associated with renal outcomes in univariate analyses.

**CONCLUSION:**

This study supports the recommendation of using the albumin-to-creatinine ratio, rather than 24-hour proteinuria, to monitor proteinuria and prognosis in primary IgA nephropathy.

## INTRODUCTION

IgA nephropathy (IgAN) is a relatively prevalent primary chronic glomerular disease occurring worldwide 1 and is the most frequently diagnosed primary glomerular disease in China 2. Proteinuria, microscopic hematuria and hypertension, either alone or in combination, are the common clinical presentations in adults 3,4. Although the clinical course is generally gradual, progression to end-stage renal disease (ESRD) is estimated to occur in 20-40% of IgAN patients within 20 years 5-7. Thus, earlier diagnoses and better monitoring of the clinical course or response to therapy are significantly important for good outcomes.

An impaired glomerular filtration rate (GFR), sustained hypertension and substantial proteinuria independently predict a poor clinical course in IgAN 4,8. Due to the lack of a disease-targeted treatment, control of proteinuria and blood pressure is emphasized by both the KDIGO and KDOQI guidelines. Therefore, it is very important that physicians monitor the levels of proteinuria and blood pressure to assess patients’ responses to therapy. Previous studies have demonstrated that time-averaged urinary protein excretion of more than 1.0 g per day indicates an extremely elevated risk of ESRD in IgAN 2,7,9. However, 24-hour urine protein collection is time consuming and often imprecise 10. Albuminuria categorization constitutes one of the chronic kidney disease (CKD) classification systems and measuring the albumin-to-creatinine ratio (ACR) in the urine has been recommended as a standard preferable to monitoring proteinuria 11,12. Many studies that have evaluated the correlation between spot urine protein levels or the ACR and 24-hour urine proteinuria (UTP) values support this methodology 13-15. However, published reports regarding the value of the ACR in predicting UTP and assessing the prognosis and progression of IgAN are rare. Therefore, the purpose of the present study was to evaluate whether the ACR is a reliable indicator of UTP in IgAN. In addition, we compared the reliability of the ACR and UTP in predicting complications of CKD and renal progression in IgAN.

## MATERIALS AND METHODS

A total of 201 inpatients with biopsy-proven IgAN were enrolled in this study from January to December 2014. The exclusion criteria were as follows: secondary causes of mesangial IgA deposition (n=16) and urinary tract infection (n=3). Of all patients evaluated, 182 satisfied the inclusion criteria of the study. Of these 182, 104 were newly diagnosed. All biopsy slides were evaluated by two pathologists using the Oxford classification criteria 16. Among these 104 patients, 44 had regular follow-up for more than 6 months and were included in the renal progression analysis.

The patients’ demographic and clinical data, such as age, gender and blood pressure, were collected during hospitalization. Therapeutic use of an angiotensin-converting enzyme inhibitor (ACEI) or angiotensin receptor blocker (ARB), prednisolone and/or other immunosuppressive agents were recorded. The main baseline characteristics of all 182 patients are shown in Table 1. The patients underwent regular visits at 3-month intervals. As with CKD, a minimal percentage change in the estimated GFR (eGFR) (25% or greater) was defined as progression 12. The follow-up time corresponded to the interval between renal biopsy and a decline in the eGFR of 25% or more compared with baseline or the last outpatient visit and patients were classified as having progression when their eGFR values decreased by 25% or more.

A first-morning void sample was collected from every patient for albumin and creatinine assessment. Each patient was then instructed to collect an accurate 24-hour urine sample for total protein quantification on the next day. Both ACR and UTP values were determined as the means of three measurements during hospitalization. To assess CKD complications, blood samples were taken on the same day as the 24-hour urine collection, 6 months later and at the last follow-up. Urine albumin and creatinine concentrations were measured via nephelometry and chromatometry, respectively, in the clinical laboratory department of our hospital. The ACR (mg/g) was calculated as the spot urine albumin concentration divided by the corresponding spot urine creatinine concentration. UTP (g/24 h) was measured using a turbidimetric method. In addition, common complications of CKD, indicated by the levels of serum albumin, hemoglobin, bicarbonate, potassium, phosphorus, total cholesterol and triglycerides, were measured. Other indexes, such as serum uric acid and cystatin C, were also measured. Adult eGFR values were calculated using the CKD-EPI equation 17, whereas the eGFR values for minors were calculated using the Filler formula 18.

This study was approved by The First Affiliated Hospital of Guangzhou University of Traditional Chinese Medicine Ethics Committees and written consent was obtained from each patient. Parents provided informed consent for minor children.

### Statistical analysis

Population characteristics are reported across the categories of CKD based on eGFR levels. Continuous data are expressed as the means ± standard deviations (SDs) or as medians, as appropriate and categorical data are expressed as numbers (percentages). The distributions of each characteristic, with the exception of age, hemoglobin, uric acid and the eGFR, were explored and found to be skewed. The Kruskal-Wallis H test was used to compare five subgroups of each baseline data set with regard to all parameters other than sex and parameters of the Oxford classification, which were compared using the Chi-square test and/or Fisher’s exact Chi-square test. Additionally, Spearman’s rank correlation was used to compare ACR and UTP values, as both of these demonstrated skewed distributions. The ability of the ACR, at various cutoffs, to predict UTP ‘thresholds’ of ≥0.15, 0.3 and 1.0 g/24 h was determined from receiver operating characteristic (ROC) curve analysis and by calculating associated sensitivities, specificities and the area under the ROC curve (AUC). An ROC curve is a graphic plot of the sensitivity *versus* (1 -specificity) for a binary classifier system. Restricted cubic splines were used to model the association between ACR and UTP values and common complications of CKD. As the placements of knots are not as important as the numbers of knots selected for restricted cubic splines (Jonas VB, Barclays UK & E RBB, 2012, unpublished data), 4 knots were determined after attempting all 3 regression runs (k=3, 4 and 5) and knot values were based on Harrell’s recommended percentiles. Knots were specifically placed at 19.95, 405.067, 1132.69 and 4619.71 mg/g for the ACR and 0.105, 0.5334, 1.4289 and 6.07 g/24 h for UTP. A canonical correlation analysis (CCA) was conducted to identify correlations between baseline proteinuria and the Oxford classification. The prognostic values of the ACR and UTP for renal progression were explored using univariate analyses and a Cox proportional hazard regression model. Variables including the ACR, UTP, age, sex, blood pressure and the CKD stage were entered into the Cox regression model. Hazard ratios (HRs) or odds ratios (ORs) and 95% confidence intervals (CIs) were calculated and significance was set at *p*<0.05 (two-sided). All analyses were conducted using Stata, version 12.0 (StataCorp LP).

## RESULTS

The baseline characteristics of all 182 participants are presented in Table 1. Among our study participants, the mean age was 33.3±12.9 years and 57.1% were female. The mean eGFR was 76.7±41.9 ml/min per 1.73 m^2^. The median spot ACR was 665.3 mg/g and the median UTP was 0.9 g/24 h. As the eGFR decreased, the ACR, but not UTP, increased. Significant differences were identified between five subgroups based on use of an ACEI or ARB, prednisolone and/or other immunosuppressive agents (*p*=0.0190). With respect to the Oxford classification, significant differences were identified among the five subgroups based on S, E and T scores. The backgrounds of 44 patients in the prospective cohort are shown in Table 2.

**Figure 1:**
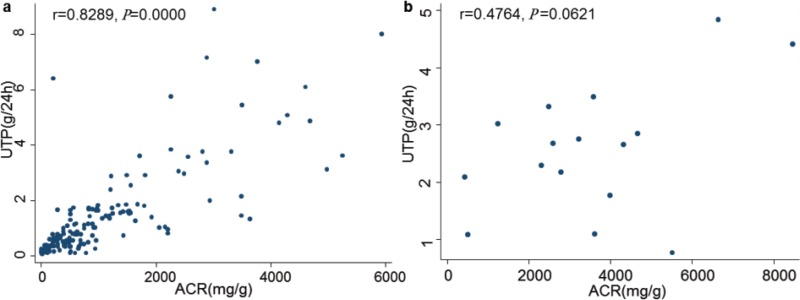
The relationship between the albumin-to-creatinine ratio and 24-hour urine proteinuria by (a) chronic kidney disease stages 1-4 and (b) stage 5.

A strong correlation was found between ACR and UTP measurements (r=0.7992, *p*=0.0000). When our analysis was stratified by subgroups based on eGFR levels, all subgroups showed a good correlation, except in CKD stage 5 (Figure 1).

**Figure 2:**
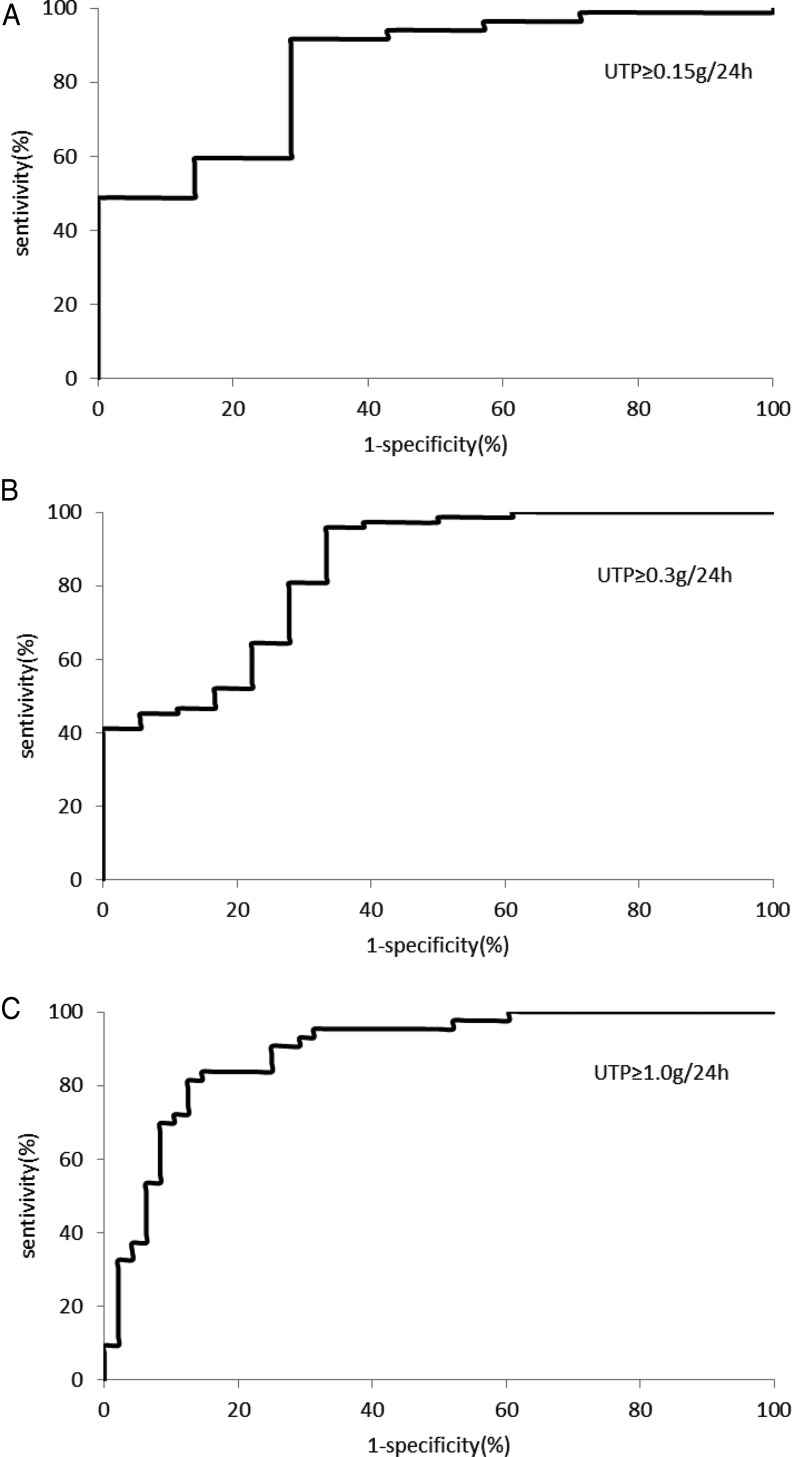
ROC curves for the albumin-to-creatinine ratio at various cutoffs to predict 24-hour urine proteinuria ‘thresholds’ of ≥0.15 (A), 0.3 (B) and 1.0 (C) g/24 h.

Using the ROC curves, ACR discriminant values of 125.15, 154.44 and 760.31 mg/g reliably predicted UTP values of ≥0.15, 0.3 and 1.0 g/24 h, respectively (Figure 2). The corresponding AUC values were 0.84, 0.84 and 0.90.

**Figure 3:**
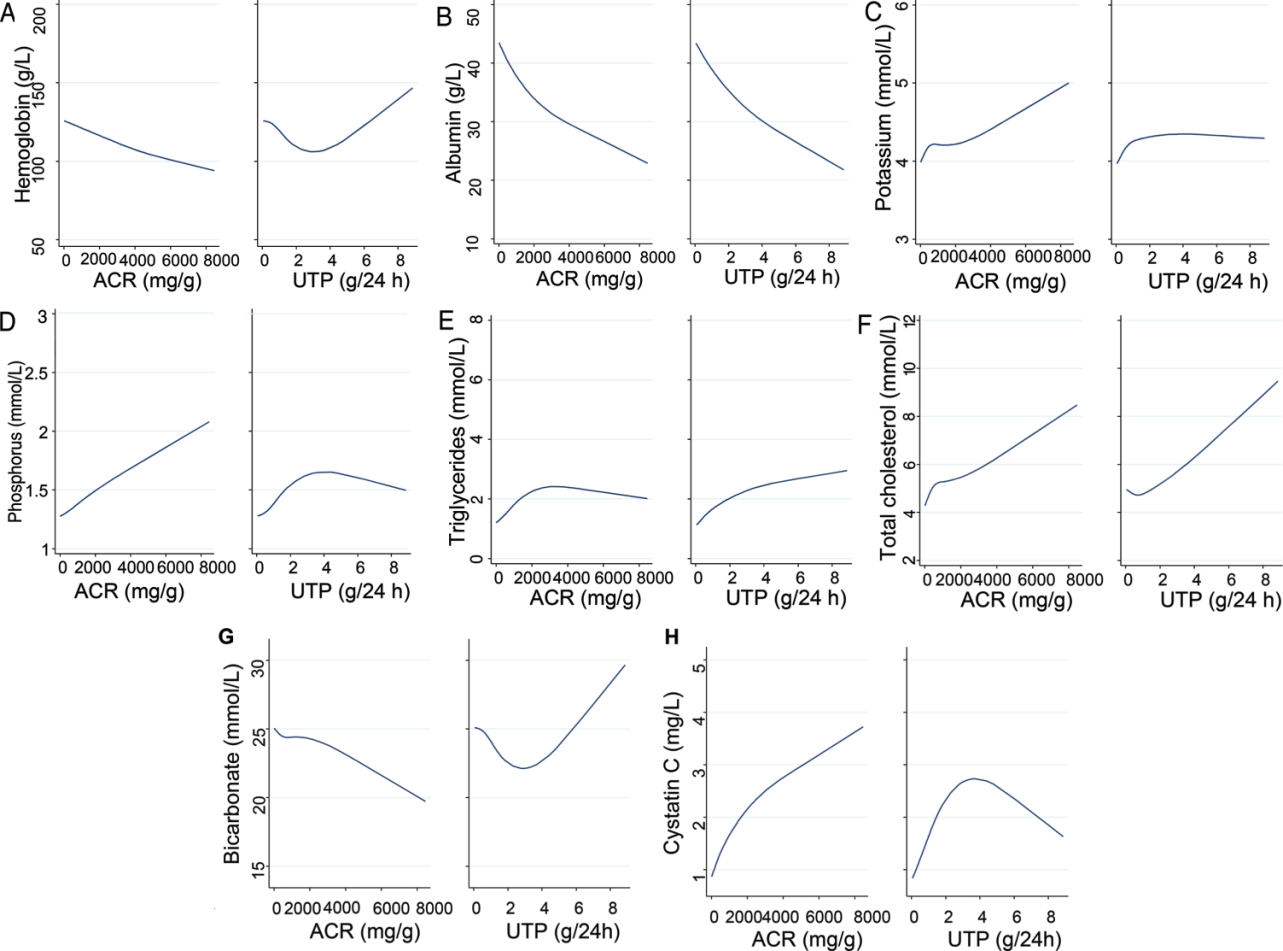
Restricted spline curves for the unadjusted association between the albumin-to-creatinine ratio and 24-hour urine proteinuria and levels of (A) serum hemoglobin, (B) serum albumin, (C) serum potassium, (D) serum phosphorus, (E) serum triglycerides, (F) serum total cholesterol, (G) serum bicarbonate and (H) serum cystatin C.

In a continuous analysis, both higher ACR and higher UTP values were associated with higher cholesterol levels (Figure 3F) and lower albumin levels (Figure 3B). Neither ACR nor UTP measurements demonstrated positive or negative correlations with uric acid levels (data not shown). Higher ACR values were associated with lower hemoglobin (Figure 3A) and bicarbonate (Figure 3G) levels and higher phosphorus (Figure 3D), potassium (Figure 3C) and cystatin C (Figure 3H) levels. However, higher levels of UTP were associated with higher hemoglobin (Figure 3A) and triglyceride (Figure 3E) levels.

To assess associations between proteinuria and the Oxford classification at the time of biopsy, a CCA model was constructed for 104 patients. This model incorporated the ACR, UTP and pathological parameters of the Oxford classification. M1, E1, S1 and T1/T2 were present in 34.62%, 3.85%, 34.62% and 15.38% of the 104 patients, respectively. CCA outputs are shown in Table 3. The canonical correlation coefficient was equal to +0.4538 (*p*=0.0016). For the proteinuria set, the ACR correlation coefficient (+0.7440) was higher than the UTP correlation coefficient (+0.3224). For the Oxford classification set, the highest canonical loading was observed for T scores (+1.1388). Thus, higher tubular atrophy and interstitial fibrosis scores were associated with greater ACR values.

The impact of the ACR and UTP on renal progression was analyzed for 44 patients. Over a median follow-up of 12 months, clinical progression was observed in 6 patients. Univariate analyses identified both the ACR (OR 8.57, 95% CI 0.80-421.80, *p*=0.0326) and UTP (OR 9.62, 95% CI 0.89-472.40, *p*=0.0229) as risk factors for eGFR decline. In the multivariate Cox regression model, neither the ACR nor UTP was a significant variable. The initial eGFR (*p*=0.044) was the only significant parameter (Table 4).

## DISCUSSION

Quantification of proteinuria is significantly important with regard to monitoring disease activity, responses to therapy and prognoses in patients with IgAN. Until recently, physicians used UTP as the gold standard for diagnosing and evaluating significant proteinuria levels. However, given that 24-hour urine collection is time consuming and often imprecise 10, other urinary protein excretion measurements are needed to complement or replace this approach. Both the KDOQI 11 and KDIGO 12 have recommended use of the protein- or albumin-to-creatinine ratio in untimed urine samples, rather than 24-hour urine collection, as the preferred method for measuring proteinuria. Certain prior studies have demonstrated a good correlation with regard to the relationship between the urine protein-to-creatinine ratio (PCR) and the UTP value in diabetic or non-diabetic patients diagnosed with renal disease. However, few studies have investigated this relationship and whether these ratios can predict the progression of CKD even more reliably than UTP measurements can in the particular case of IgAN.

In the present study, we found a strong correlation between ACR and UTP values throughout the overall study population. Except in the CKD stage 5 group, good correlations were also observed when we divided the patients into five subgroups based on eGFR levels. A previous study showed that renal function has little influence on the urine PCR 19. Moreover, proteinuria declines with glomerulosclerosis and advanced stages of CKD exhibit disturbed circadian blood pressure rhythms 20, structurally deficient arterioles 21 and eGFR variability 22. Whether the exception described above is due to the reasons discussed here remains unknown. However, these findings are in agreement with a published report 23.

In clinical settings, the UTP ‘threshold’ is defined to evaluate the degree of kidney injury. Usually, a UTP value less than 0.3 g/24 h is a criterion for complete remission in IgAN patients. However, higher levels of proteinuria (>3.5 g/24 h) indicate severity in the nephrotic range and are associated with faster progression of kidney disease and increased risks of cardiovascular disease 24. In the present study, the discriminant values required for spot urine ACR values to predict UTP values at these ‘threshold’ levels were calculated with excellent sensitivity, specificity and AUC values. In general, a first-morning urine ACR ≥125.15, 154.44 or 760.31 mg/g should reliably predict UTP values ≥0.15, 0.3 and 1.0 g/24 h, respectively. These results are slightly different from the values cited in the KDIGO guidelines 12. Furthermore, in clinical practice, UTP <0.15 g/24 h and/or an ACR <30 mg/g are defined as normal, but in our study, ACR values ≥125.15 mg/g were equivalent to UTP values ≥0.15 g/24 h. The observation that ACR values ≥760.31 mg/g were equal to UTP values ≥1.0 g/24 h also differed from the result of a prior study 23; this discrepancy may be due to the cause of CKD. These discriminant spot ACR values are applicable to monitoring clinical courses and responses to therapy. Based on previous reports 11,12,14, a random urine aliquot is just as accurate as a first-morning urine specimen in predicting proteinuria and albuminuria. Experiments investigating whether our findings can be extrapolated to random urine collections are currently underway.

To our knowledge, few prior studies have evaluated the performance of different proteinuria measurements in predicting renal events. A study of 98 non-diabetic outpatients with chronic renal disease found that PCR and UTP values similarly predicted ESRD when used in univariate analyses but that the PCR was the only parameter for which multivariate analyses demonstrated statistical significance 24. However, proteinuria is not the same as albuminuria. Another study of 700 diabetic patients found that compared with albuminuria or proteinuria detected using 24-hour urine specimens, the ACR was more strongly associated with the risk of ESRD or doubling of creatinine levels 25. As the pathological changes associated with different renal diseases are distinct, whether these findings can be extrapolated to non-diabetic patients is not clear. Here, in a cross-sectional study, we showed that ACR values were significantly associated with anemia, acidosis, hypoalbuminemia, hyperphosphatemia, hyperkalemia, hypercholesterolemia and higher serum cystatin C. However, higher UTP values were only associated with hypoalbuminemia and hypercholesterolemia. Thus, ACR measurements displayed a stronger and more stable association with CKD complications in IgAN. Cystatin C is a 13-kDa basic protein of the cystatin superfamily of cysteine proteinase inhibitors that is a more sensitive biomarker than serum creatinine in monitoring renal function 26. In the current study, as the eGFR declined, serum cystatin C increased. Moreover, according to the baseline characteristics in this study, as the eGFR declined, the ACR, but not UTP, increased. A previous study also observed a good correlation between serum cystatin C and renal histology, particularly with regard to interstitial cellular infiltration and tubular atrophy in patients with IgAN 27. In agreement with these results, our study also showed a better correlation between the ACR and tubulointerstitial changes than between UTP and these changes. As IgAN is not a disease with benign progression, managing and preventing metabolic complications of CKD are vital to postponing ESRD.

In addition to our cross-sectional analysis, we performed a prospective observational study to investigate the influence of the ACR and UTP on long-term renal progression. By univariate analysis, ACR and UTP values were identified as the clinical risk factors that predicted ≥25% of eGFR decreases. However, our multivariate analyses indicated that neither the ACR nor UTP influenced renal progression. These findings could be explained by the small follow-up sample size, the use of treatment with immunosuppressive agents or renin-angiotensin-aldosterone system inhibitors 28 and the strong predictive effect of the baseline eGFR.

Our study also provides interesting data regarding the correlation between proteinuria and pathological parameters of the Oxford classification in renal biopsy. Many previous studies have indicated that tubular atrophy and interstitial fibrosis, as indicated via initial renal biopsy, predict adverse renal outcomes in patients with IgAN 29,30. Interestingly, we found that the presence of T1/T2 was significantly associated with higher ACR, but not UTP, measurements. Previous studies have shown that proteinuria is a rigorous predictor of tubulointerstitial disease progression and the decline of renal function 31,32. A prospective study 33 enrolled 215 patients with CKD, among whom 43 patients presented IgAN, to assess the relationships between ACR levels, urinary monocyte chemoattractant protein-1 (MCP-1) levels, macrophage numbers, *in situ* damage and clinical outcomes. In subset analyses classified by histological diagnosis, ACR measurements correlated with interstitial macrophage numbers only in the cases of IgAN and focal segmental glomerulosclerosis. Moreover, in the early and middle stages of CKD, the proportions of total renal creatinine excretion due to tubular secretion are fairly constant within a given patient. However, total urine creatinine excretion actually declines because extrarenal creatinine degradation increases with declining renal function 26. For these reasons, the ratio of the concentrations of urinary albumin and creatinine in a single voided urine sample cancels out the time factor and correlates well with GFR decline and tubulointerstitial changes.

The present study is unique in that it combined cross-sectional and prospective follow-up data to evaluate the utility of the ACR in monitoring renal progression. Previous studies have shown that lead-time bias may limit estimations and comparisons of kidney survival in IgAN patients when only initial data are considered 34,35. In addition, the Oxford classification was employed to explore associations between pathological and clinical indexes in the current study. Finally, all laboratory indexes were measured in a single clinical laboratory department. In addition to the common complications observed with regard to CKD, uric acid and blood lipids are frequently observed to rise in IgAN patients; thus, these two parameters were included in our analyses.

This study is limited by its relatively small size and short follow-up period. In addition, the study participants demonstrating eGFR levels <15 ml/min per 1.73 m^2^ were fewer than those in the other subgroups. Finally, because this was an observational study, treatments differed based on different physician preferences, such that we were unable to examine therapeutic effects.

In conclusion, we have demonstrated a good correlation between spot ACR and UTP values, except those pertaining to CKD stage 5, in IgAN patients. Spot urine ACR values of ≥125.15, 154.44 and 760.31 mg/g reliably predicted equivalent UTP ‘threshold' values of ≥0.15, 0.3 and 1.0 g/24 h, respectively. ACR measurements were significantly associated with anemia, acidosis, hypoalbuminemia, hyperphosphatemia, hyperkalemia, hypercholesterolemia and higher cystatin C levels. However, the association of UTP values with these concurrent complications was not consistent. Baseline ACR values demonstrated a stronger association with the Oxford classification and correlated with renal progression. Analyses of first-morning void samples and ACR measurements appear to constitute a superior means of evaluating and managing CKD complications compared with UTP measurements. Thus, spot ACR measurements constitute a precise standard by which proteinuria can be measured and are more reliable than 24-hour urine protein specimens in predicting progression in IgAN.

## AUTHOR CONTRIBUTIONS

Yuezhong L was responsible for the study design. Huan L was responsible for the statistics and manuscript writing. Chao W and HaiTao T were responsible for the included cases.

## Figures and Tables

**Table 1:** Characteristics of the study population, stratified by eGFR level.

Characteristic	Total	Stages of CKD					*p-*value
		Stage 1	Stage 2	Stage 3	Stage 4	Stage 5	
No. of participants	182	74	44	32	16	16	—
Age (y)	33.3±12.9	26.1±10.6	38.5±10.8	39.6±13.2	31.6±9.5	41.1±13.8	0.0001
Female	57.1	51.4	68.2	56.3	62.5	50.0	0.4430
Serum creatinine (μmol/L)	166.2±203	70.9±14.3	88.0±15.3	155.6±32.8	285.5±37	723.6±286.7	0.0001
Uric acid (μmol/L)	427.2±123.4	366.1±96.6	386.6±73.1	530.9±112.5	547.9±83.3	516.8±145	0.0001
eGFR (ml/min/1.73 m^2^)	76.7±41.9	116.5±20.5	80.2±7.5	42.4±8.5	20.7±4.0	7.5±3.1	0.0001
Cystatin C (mg/L)	1.6±1.1	0.9±0.2	1.1±0.3	1.9±0.7	3±0.3	4.3±0.6	0.0001
ACR (mg/g)	665.3 [511.1-837.3]	295.9 [214.7-409.4]	792.0 [510.6-967.1]	715.6 [515.9-966.5]	2044.0 [921.2-3386.0]	2910.4 [2405.2-4644.0]	0.0001
UTP (g/24 h)	0.9 [0.7-1.1]	0.4 [0.3-0.6]	0.8 [0.6-1.3]	0.9 [0.7-1.4]	2.4 [1.3-5.2]	2.7 [1.7-3.1]	0.0001
MAP (mmHg)	95.5±16.7	85.7±8.9	95±14.8	98.2±8.4	116.2±26	115.7±13.1	0.0001
Bicarbonate (mmol/L)	24.4±3.4	25.3±2.3	25.9±2.8	24.3±2.5	21.6±5.2	19.7±2.9	0.0001
Phosphorus (mmol/L)	1.4±0.3	1.3±0.2	1.2±0.2	1.4±0.2	1.5±0.2	2.1±0.3	0.0001
Potassium (mmol/L)	4.2±0.5	4±0.4	4.1±0.4	4.3±0.4	4.6±0.6	4.7±0.5	0.0001
Hemoglobin (g/L)	120.3±24.2	130.1±17.6	126.3±22.8	116±18.2	105.5±23	81.8±20.1	0.0001
Albumin (g/L)	37.8±7.2	38.8±8.5	38.6±7.0	38.0±4.6	34.2±4.6	34.8±5.9	0.0001
Triglycerides (mmol/L)	1.7±1.2	1.3±0.8	1.7±1.1	2.5±1.8	2.2±1.0	1.7±1.0	0.0001
Cholesterol (mmol/L) Oxford classification	5.2±1.9	5.4±2.4	5.1±1.7	5.4±1.7	5.6±1.1	4.6±1.1	0.1556
M(0/1)	116/66	54/20	26/18	17/15	12/4	7/9	0.0800
S(0/1)	108/74	52/22	32/12	13/19	6/10	5/11	0.0010
E(0/1)	167/15	74/0	41/3	28/4	14/2	10/6	0.0000
T(0/1/2)	142/34/6	74/0/0	37/7/0	22/10/0	8/8/0	1/9/6	0.0000
Therapy	64.8	54.1	63.6	87.5	62.5	75.0	0.0190

Values are percentages, numbers, means ± SDs or medians [95% CI]. Therapy includes immunosuppressive agents and ACEI or ARB use.

MAP = mean arterial pressure; M = mesangial hypercellularity; S = segmental glomerulosclerosis; E = endocapillary hypercellularity; T = tubular atrophy and interstitial fibrosis.

**Table 2:** Characteristics of patients in the prospective cohort, stratified by eGFR level.

Characteristic	Total	Stages of CKD					*p*-value
		Stage 1	Stage 2	Stage 3	Stage 4	Stage 5	
No. of participants	44	19	15	8	2	0	—
Age (y)	31.9±11.8	26.5±10.4	38.5±10.6	35.3±10.6	21±11.3	—	0.0111
Female	63.6	52.6	73.3	75.0	50.0	—	0.5300
Serum creatinine (μmol/L)	99.3±53.2	71.1±16.2	84.8±12.7	147.3±35.1	283.5±37.5	—	0.0001
Uric acid (μmol/L)	420±121.2	357.8±99.7	406.6±79.6	572.3±91.1	502±176.8	—	0.0011
eGFR (ml/min/1.73 m^2^)	86.9±33.2	115.2±21.3	82.1±5.1	44.7±10.2	22.9±0.9	—	0.0001
Cystatin C (mg/L)	1.2±0.6	0.8±0.2	1.1±0.3	1.7±0.6	2.9±0.6	—	0.0001
ACR (mg/g)	512.5 [329.4-836.3]	218.1 [59.0-564.1]	760.3 [329.4-1181.4]	715.6 [346.0-2384.0]	2457.2 [1135.8-3778.6]	—	0.0260
UTP (g/24 h)	0.8[0.6-1.3]	0.5[0.2-1.2]	1.1[0.7-1.6]	0.7[0.3-3.6]	4.2[1.4-7.0]	—	0.1731
MAP (mmHg)	92.0±16.3	84.7±10.0	93.9±13.5	100.0±9.4	115.7±60.8	—	0.0205
Bicarbonate (mmol/L)	25.3±2.3	25.4±2.0	25.9±3.1	23.8±1.0	25.1±0.1	—	0.1419
Phosphorus (mmol/L)	1.3±0.2	1.3±0.2	1.2±0.2	1.4±0.2	1.8±0.0	—	0.0522
Potassium (mmol/L)	4.1±0.4	3.9±0.4	4.0±0.4	4.5±0.3	4.5±0.6	—	0.0077
Hemoglobin (g/L)	122.2±18.6	126.3±18.2	122.5±16.9	118±20.2	97.5±19.1	—	0.2137
Albumin (g/L)	37.9±7.0	39.4±7.3	36.8±7.6	38.1±4.2	30.7±4.5	—	0.1090
Triglycerides (mmol/L)	1.7±1.2	1.2±0.5	2.0±1.2	2.1±1.8	3.0±1.4	—	0.0105
Cholesterol (mmol/L)	5.2±1.9	4.8±1.5	5.1±1.8	5.5±1.5	6.2±1.3	—	0.3044
Oxford classification	35/9	17/2	11/4	5/3	2/0	—	0.3270
M(0/1)	32/12	15/4	11/4	4/4	2/0	—	0.3610
S(0/1)	42/2	19/0	14/1	8/0	1/1	—	0.0120
E(0/1)	40/4/0	19/0/0	13/2/0	7/1/0	1/1/0	—	0.0940
Therapy	54.5	42.1	46.7	87.5	100.0	—	0.0810

**Table 3:** Standardized coefficients for the proteinuria and Oxford classification variables.

	Proteinuria variables		Oxford classification variables			
Characteristic	ACR	UTP	M	S	E	T
Standardized coefficient	+0.7440	+0.3224	-0.2883	+0.0870	-0.5079	+1.1388

**Table 4:** Cox regression model for eGFR decline.

Variable	Value	HR	95% CI	*p*-value
ACR	0/1	2.0025	0.65-6.12	0.223
UTP	0/1	0.3884	0.13-1.14	0.084
Age	years	0.9992	0.96-1.04	0.971
Sex	0/1	2.1668	0.91-5.18	0.082
CKD stage	1/2/3/4/5	1.6190	1.01-2.59	0.044
Blood pressure	0/1	0.4347	0.12-1.61	0.213
